# EHMTI-0093. Investigation of 5-HT2B receptor pathways with relevance to a mouse migraine model

**DOI:** 10.1186/1129-2377-15-S1-F13

**Published:** 2014-09-18

**Authors:** M Kremser, A Hunfeld, H Lübbert

**Affiliations:** 1 Department of Animal Physiology, Faculty of Biology and Biotechnology, Bochum, Germany

## Introduction

Recent research raises the question whether the serotonin 2B receptor (5-HT2B) plays a role in the pathogenesis of migraine. Clinical studies revealed that the 5-HT2B/2C agonist meta-Chlorophenylpiperazine (mCPP) induces migraine-like headache more likely in migraineurs than in subjects without a history of migraine. We therefore developed an animal model for chronic migraine, where we are able to induce a neurogenic inflammation in the dura mater of hypoxic mice with 5-HT2B agonists. This inflammation can be blocked by specific 5-HT2B inhibitors.

Until now little is known about the 5-HT2B receptor: It is expressed on endothelial cells of blood vessels, but it may also be present on other cell types. Like most of the other serotonin receptors it is a G protein-coupled receptor, but the native signal transduction pathway after receptor activation is not clear yet.

## Aims

Investigation of the 5-HT2B receptor in a primary cell culture system to determine native signal transduction pathways.

## Methods

Cultivation of primary cells. Validation of the presence of the receptor. Signal transduction assays.

## Results

Stimulation with the 5-HT2B/2C agonist mCPP induced concentration-dependent ERK phosphorylation in 5-HT2B positive primary cells.

## Conclusions

Activation of the 5-HT2B receptors may stimulate cell proliferation and angiogenesis and therefore alter the vascular system of the dura mater, which may result in a higher susceptibility for migraine.

No conflict of interest.

